# Exploring the relationship between the speed-resolved perfusion of blood flux and HRV following different thermal stimulations using MSE and MFE analyses

**DOI:** 10.1371/journal.pone.0217973

**Published:** 2019-06-05

**Authors:** Guangjun Wang, Shuyong Jia, Hongyan Li, Xiaojing Song, Weibo Zhang

**Affiliations:** Institute of Acupuncture and Moxibustion, China Academy of Chinese Medical Sciences, Beijing, China; University of Illinois at Urbana-Champaign, UNITED STATES

## Abstract

Our previous study employed the classic laser Doppler flux (LDF) to explore the complexity of local blood flow signals and their relationship with heart rate variability (HRV). However, microcirculation blood flow is composed of different velocity components. To investigate the complexity of local speed-resolved perfusion and HRV following stimulation with different temperatures in healthy subjects, multiscale entropy (MSE) and multiscale fuzzy entropy (MFE) were used to measure the complexity of local speed-resolved perfusion signals. MSE was also used to evaluate the complexity of HRV. The results indicated that thermal stimulation increased all components of local speed-resolved perfusion and that stimulation with different temperatures resulted in different changes in the complexity area index. However, the same stimulation had no effect on the MSE of HRV. Further research showed that 44°C thermal stimulation resulted in a weak correlation between the composite speed-resolved perfusion and the HRV complexity. The current study provides a new approach for studying the relationship between speed-resolved perfusion signals and cardiac function.

## Introduction

There is increasing evidence that microcirculation can be used to evaluate vascular disorders at the systemic level[1, 2], and there is a close relationship between cardiac and vessel functions[3, 4]. In general, the functional status of vessels can be assessed by laser Doppler flowmetry (LDF). However, it is difficult to differentiate between different vascular compartments using the classical LDF approach. Recently, a multiparameter model based on the Monte Carlo algorithm has provided the possibility of further distinguishing the different velocity components in microcirculation perfusion[5–8]. This new method may provide further insight into evaluating vascular dysfunction at the systemic level[9, 10].

It has been accepted that circulatory system regulation is a nonlinear process[11]. From this perspective, a classic spectrum analysis of laser Doppler flux is unable to describe the dynamic characteristics of blood flux[11]. Previous studies have shown that nonlinear dynamic analysis can provide information about the variability of skin blood flow oscillations[11, 12], and sample entropy (S_E_) has been used to evaluate the skin blood perfusion flux (SkBF) response resulting from thermal stress[13]. In particular, MSE analysis provides a more powerful method for complex measurements[14, 15] of vascular dynamics. MSE has also been used to analyze the LDF time series[16–18]. In short, MSE quantifies the degree of irregularity of a time series on multiple time scales. Because of the highly irregular time series in a wide range of time scales, time series with larger entropy are considered to be more complex than those with irregular behavior only on a single time scale[19]. In our previous study, both multiscale entropy (MSE) and multiscale fuzzy entropy (MFE) were used to assess the skin blood perfusion response to thermal stimulation[18]. Considering that MFE has a more significant correlation with MSE than do other multiscale entropies[20], both MSE and MFE were analyzed in the previous[18] and current study. MSE analysis consists of three steps in the current study: (1) coarse graining of the speed-resolved blood flux to obtain multiple signals, each of which captures the system dynamics at a given scale; (2) calculating the SampEn and fuzzy entropy for speed-resolved blood flux; and (3) integrating the entropy values within the pre-defined scale to obtain the complexity area index[19, 21].

Heart rate variability (HRV) analysis is generally a useful method to evaluate autonomic functions[22]. Previous studies have indicated that temperature stress significantly modulates HRV[23, 24]. Methodologically, a nonlinear analysis of heart rate may provide more information than time- or frequency-domain results of HRV[25]. The MSE of HRV could be used as a potential index to distinguish healthy subjects from patients with atrial fibrillation[15]. Our previous studies suggested that both the MSE and MFE of LDF vary significantly under different thermal stimulations, particularly under 42°C or 44°C stimulation. Both the MSE and MFE of LDF are moderately correlated with the complexity of HRV. The current study further analyzed the complexity of LDF and its relationship with HRV based on the speed-resolved blood flux.

## Materials and methods

### Inclusion and exclusion criteria

Healthy subjects aged 18 to 60 years were included. Alcohol, tea and coffee intake were prohibited for at least 24 hours prior to measurements. None of the subjects were taking any medication that would affect cardiovascular or autonomic regulation.

### Participants and design

**Fig 1:**
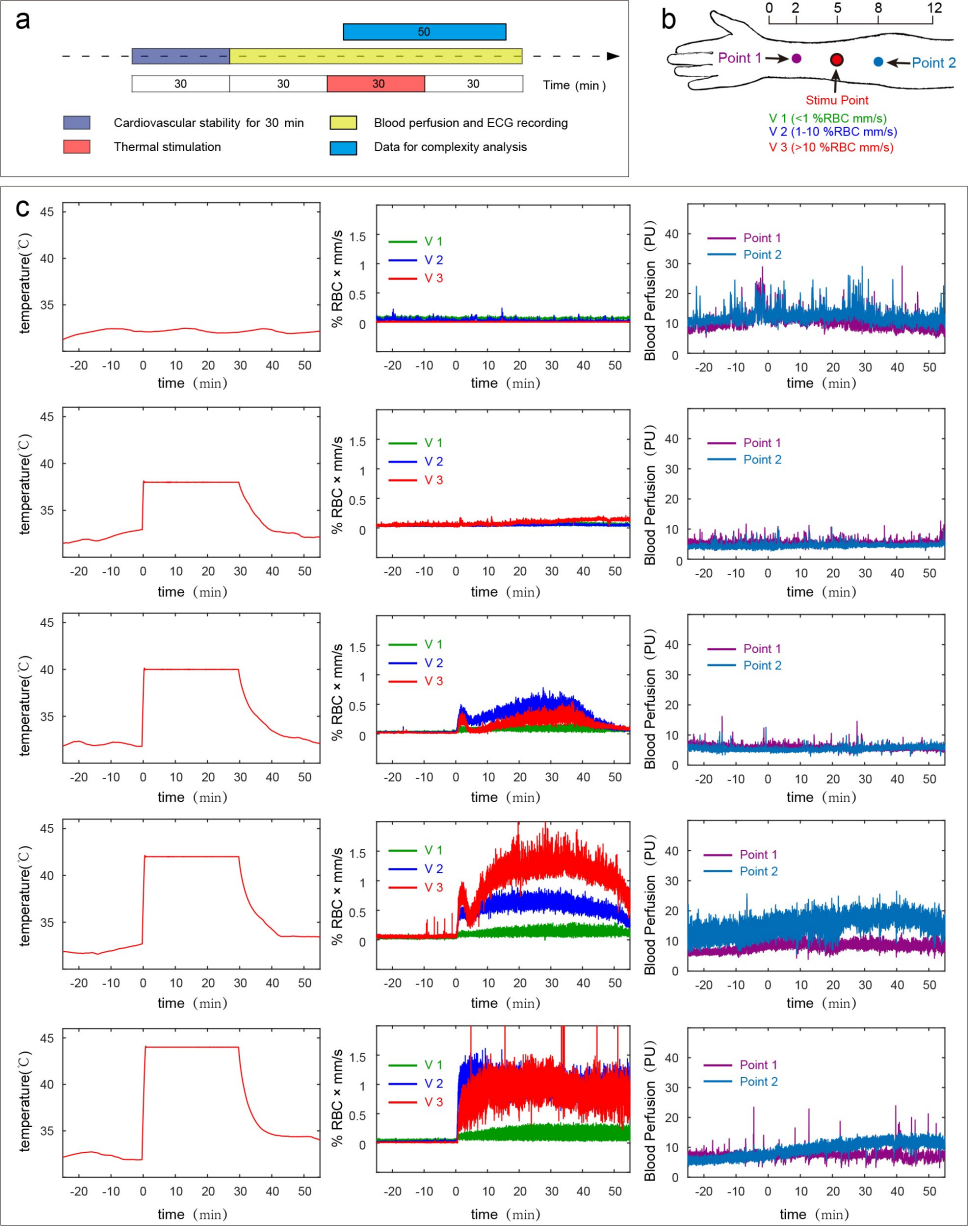
Experimental design and case subject raw data. (a) Experimental design. (b) Thermal stimulation and recording point locations. The thermal stimulation point was located on the anterior aspect of the forearm, between the tendons of the palmaris longus and the flexor carpi radials, 5 B-cun proximal to the palmar wrist crease. Control point 1 was located 2 B-cun proximal to the palmar wrist crease, and control point 2 was located 8 B-cun proximal to the palmar wrist crease[30]. (c) Temperature (left), speed-resolved perfusion signal (middle) and blood perfusion signals at the control points (right). V1, low speed, <1 mm/s; V2, mid-speed, 1–10 mm/s; V3, high speed, >10 mm/s.

A total of 60 healthy subjects were recruited, and all of them completed the measurements and were included in the statistical analysis. All subjects completed measurements between 9 a.m. and 5 p.m. Detailed information about the participants is presented in Table 1. In the current study, subjects in the thermal stimulation group received a total of 4 times the stimuli corresponding to 38°C, 40°C, 42°C and 44°C. The order of thermal stimulation was randomly determined for each subject, which is provided in S1 Table. The experimental protocol was as follows (Fig 1A): All experiments were carried out in a quiet, temperature-controlled (24–26°C) laboratory. After a period of cardiovascular stability (30 min), a baseline recording was made for 30 min. Then, the test subjects were stimulated by using a thermal stimulation PF 6000 unit (Perimed AB, Stockholm, Sweden) for 30 min followed by a 30-min rest period. In the blank control group (BC group), the measurement process was the same as that in the temperature stimulation group (TS group), except that the probe was not heated to keep the subjects in a resting state so that the blood flow parameters of the subjects could be obtained in a resting state.

**Table 1:** Subject characteristics.

	BC group			TS group			t/χ^2^	*p*
	Max	Min	Mean±SD	Max	Min	Mean±SD		
Age (years)	32	23	26.6±2.06	49	23	26.7±4.86	0.104	0.918
Height (cm)	175	155	162.23±5.7	185	155	166.6±7.47	2.545	0.014
Weight (kg)	79	45	55.8±8.67	100	44	61.4±13.17	1.946	0.057
BMI	36.28	13.06	19.51±5.88	54.64	12.1	23.34±9.71	1.850	0.069
Gender	3M/27F			8M/22F			2.783	0.095

BC, blank control; TS, thermal stimulation.

### Protocol for blood perfusion measurement and analysis

During the recording session, subjects were placed in a supine position, and their forearms were fixed with a vacuum pillow (AB Germa, Kristianstad, Sweden). Microcirculation was measured by a PeriFlux 6000 Enhanced Perfusion and Oxygen Saturation (EPOS; Perimed AB, Stockholm, Sweden) system with a 3-Hz sampling rate according to the previously described recording protocol[9, 26]. All of the measurement processes were set up in the EPOS management system. During measurements, the EPOS flat probe was fixated using double-sided adhesive tape (PF 105–1, Perimed AB, Stockholm, Sweden). The EPOS System was used for a multimodal assessment of the microcirculation using diffuse reflectance spectroscopy (DRS) and laser Doppler flowmetry (LDF). Using the EPOS system, perfusion of the stimulated point was evaluated for three speeds (V1, <1 mm/s; V2, 1–10 mm/s; V3, >10 mm/s). In this study, the probe combined both a laser Doppler probe and a thermostatic probe at the stimulated point, which allowed for a controlled, consistent heating of the skin area under the probe surface. The classic LDFs of both control points (points 1 and 2) were recorded by a PeriFlux 5000 (Perimed AB, Stockholm, Sweden) system with a 64-Hz sample rate. The method used to record and analyze the blood perfusion flux signal is described in our previous studies[27–29]. Both thermal stimulation points and control points are shown in Fig 1B. The temperature recordings, speed-resolved perfusion and classic LDF of the control points for one case subject are shown in Fig 1C. For the speed-resolved perfusion results recorded by PF6000, the data were exported directly from the EPOS system in MATLAB format and then were imported into MATLAB software and analyzed; for the results, which were recorded by PF5000, the data were opened with PeriSoft for Windows (version 2.5.5, Perimed, Sweden) and then were exported in the txt format. Finally, the data were imported into MATLAB and were analyzed. At each time point, the mean blood flow of one minute (30 seconds before and 30 seconds after) was calculated.

### Complexity of the blood flux signal

The complexity of the blood flux signal was evaluated by MSE and MFE. The theory of MSE is described by Costa[14, 15], and the analytical methods of the MATLAB toolbox are provided by PhysioNet[31]. MFE was calculated using the MATLAB toolbox provided by Azami, H[32]. A total of 50 min of blood perfusion signals was used for the MSE and MFE analyses, and the complexity area index was calculated by plotting the sample entropy of each coarse-grained time series and then the area was calculated under the given curve[21, 33]. In the current study, 20 scale factors and the X-axis make up 19 trapezoids. After calculating the area of each trapezoid, the sum of these areas is the area under the curve. The analysis parameters were described in a previous study (for MSE, m = 2, r = 0.15; for MFE, m = 2, r = 0.15, fuzzy power = 2)[18]. The analysis method for MSE and MFE is described in S1 File.

### Electrocardiogram measurement protocol and MSE analysis

The ECG was measured by the NeurOne system (NeurOne, MEGA electronics Ltd, Finland). Subjects lay on a comfortable bed, and 3 adhesive Ag/AgCl electrodes (3M, Shanghai, China) were applied to the chest for ECG recording at a sampling rate of 1000 Hz [27, 34–36]. The raw data were imported into Kubio HRV software (Kubios HRV Premium 3.0.0, Kubios oy, Kuopio, Finland) in the ASC format, and then the heart rate MSE was analyzed [37], which is described in S1 File. The complexity area index was also calculated.

### Statistical analysis

Data are presented as the mean±SE. A permutation test analysis was used to assess the differences among the control points and temperatures. The correlation of the complexity area index between blood perfusion and HRV was analyzed using Spearman’s correlation coefficient (SCC). Mixed repeated-measurement ANOVA was calculated to analyze between-subject factors with SPSS software 23.0 (IBM SPSS Inc, Chicago, IL, USA), and a permutation test was used for posttests. Multiple comparisons with the BC group were performed as part of an independent design, while multiple comparisons between the different stimuli were performed as pairwise. All permutation test analyses were conducted using MATLAB software 2015b (MathWorks, Natick, Massachusetts, USA). All reported *P* values were two-sided, and the level of significance was defined as *P*<0.05.

### Ethical approval and consent to participate

Written informed consent for thermal stimulation and for the use of the data of this study was obtained from each patient. The written consent was approved by the Institutional Research Ethics Boards of Acupuncture & Moxibustion of the China Academy of Chinese Medical Sciences.

## Results

A total of 60 subjects were recruited in the current study. Detailed information on the participants receiving thermal stimulation (TS group, n = 30) and the blank control subjects (BC group, n = 30) is summarized in Table 1. In the thermal stimulation group, the stimulation order was randomly generated for each subject, which is detailed in S1 Table. The experimental design is shown in Fig 1A. The blood flux recording positions are shown in Fig 1B. The different thermal stimulation conditions, the related speed-resolved perfusion and the classic LDF are shown in Fig 1C. The time for each subject to participate in the measurement was not fixed in the current study. To exclude the influence of circadian rhythm from the analysis results, we compared the heart rates of the subjects before each intervention, and there was no significant difference between the groups (S2 Table).

**Fig 2:**
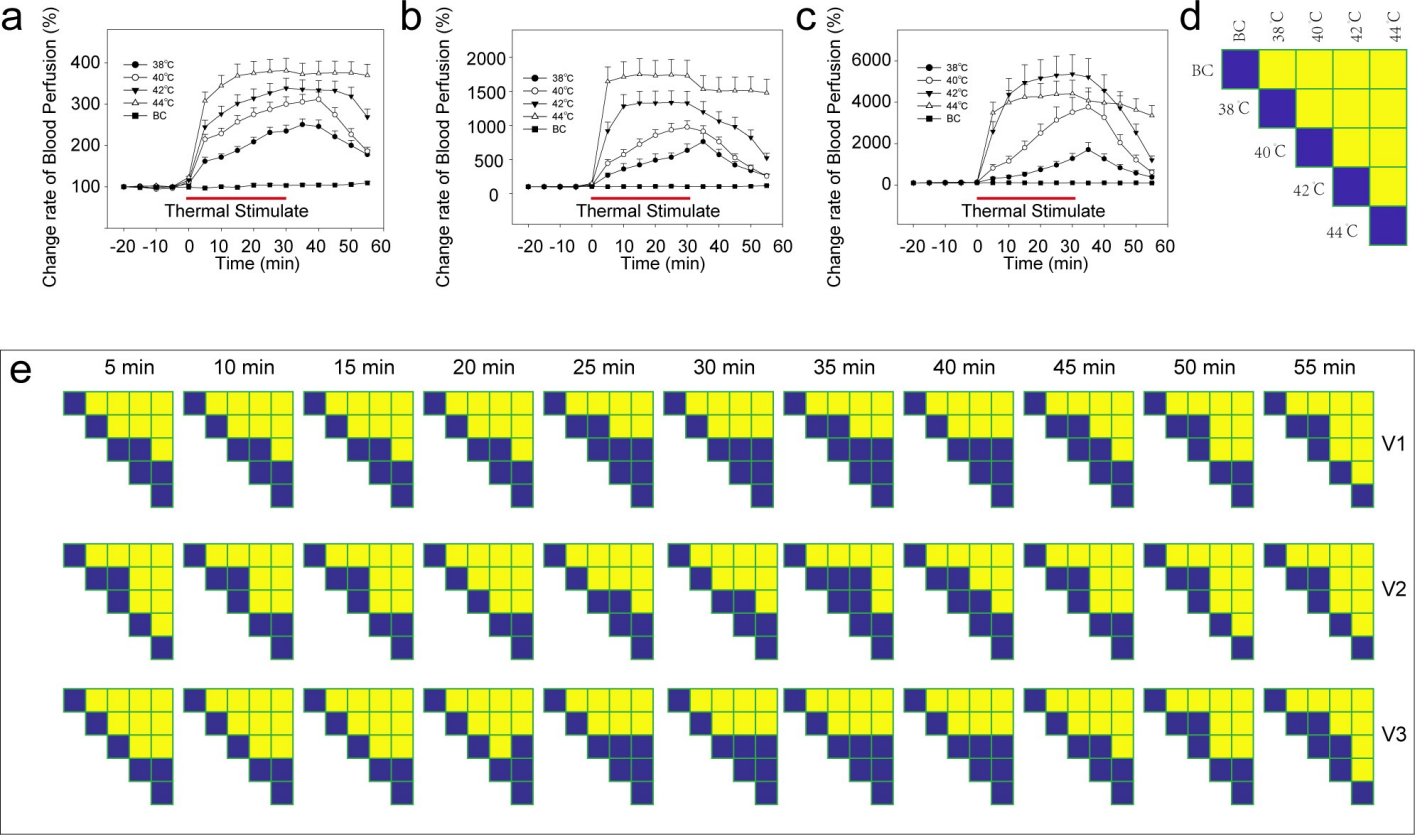
Speed-resolved perfusion at the stimulation point. (a) Low-speed component (V1, <1 mm/s), (b) Mid-speed component (V2, 1–10 mm/s). (c) High-speed component (V3, >10 mm/s). (d) Design of the comparison of speed-resolved perfusion at each time point. (e) Results of the comparison of the speed-resolved perfusion at each time point. Repeated measures ANOVA was calculated to analyze the between-subject factor, (V1: *F*_(4,145)_ = 34.05, *P*<0.0001; V2: *F*_(4,145)_ = 23.66, *P*<0.0001; V3: *F*_(4,145)_ = 16.638, *P*<0.0001), and permutation tests (two-sided, 1000 permutations) were used as posttests. Yellow indicates P<0.01, and blue indicates P>0.01. BC, blank control; Data are presented as the mean±SE.

**Fig 3:**
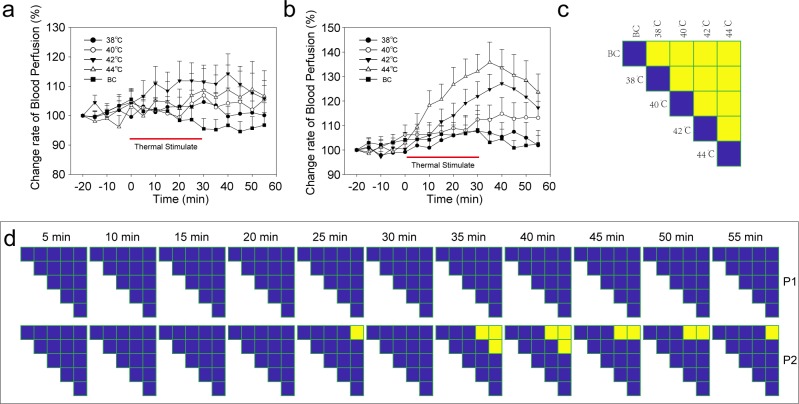
Blood perfusion at control points. (a) Control point 1. (b) Control point 2. (c) Design of the comparison of blood perfusion at each time point. (d) Results of the comparison of blood perfusion at each time point. Repeated measures ANOVA was calculated to analyze the between-subject factor, (P1: *F*_(4,145)_ = 0.796, *P* = 0.529; P2: *F*_(4,145)_ = 3.028, *P* = 0.02), and permutation tests (two-sided, 1000 permutations) were used as posttests. Yellow indicates P<0.01 and blue indicates P>0.01, permutation test (two-sided, 1000 permutations). P1, control point 1; P2, control point 2; BC, blank control; Data are presented as the mean±SE.

The temporal results for the different velocity components during and after thermal stimulation are shown in Fig 2. The blood flux of each speed-resolved perfusion component significantly increased after 5 min of stimulation (Fig 2A, Fig 2B, Fig 2C and Fig 2E). For each speed-resolved component under 38°C or 40°C stimulation, the blood flux increased as the stimulation was prolonged. The blood flux decreased 5 minutes after cessation of stimulation. However, under 42°C or 44°C stimulation, the patterns of the blood flux signal were completely different among the distinct velocity components. Under stimulation of 42°C, the low-velocity component (V1) increased with the prolongation of stimulation and was then maintained at a certain level (Fig 2A). The mid-velocity component (V2) reached the maximum at 10 min after stimulation and began to decline at the end of the stimulus (Fig 2B); however, the high-velocity component (V3) first increased and then decreased in a parabolic pattern (Fig 2C). However, for 5 min of 44°C (Fig 2D) stimulation, all components were near the maximum, and the blood flow was maintained at this level with no significant attenuation. Unlike control point 1 (Fig 3A), control point 2 (Fig 3B) showed increased blood flow at 42°C or 44°C stimulation.

To determine the local effect of blood perfusion following thermal stimulation, the time series of different speed-resolved components were analyzed using a complexity analysis. In the current study, both the MSE and MFE were evaluated. To evaluate the reliability of the methods, as in our previous study[18], both the MFE and the MSE of white noise were also calculated (S1 Fig), which indicated that our methods and parameters were suitable[14, 15]. Our previous study recorded a conventional LDF at a 64-Hz sampling rate; however, the maximum sampling that can be achieved by the Enhanced Perfusion and Oxygen Saturation (EPOS) system in the current study is 3 Hz. Therefore, we also analyzed the effect of different sampling rates on the complexity of white noise signals. The results suggest that for white noise signals, the sampling rates between 3 Hz and 64 Hz do not affect the signal complexity or the complexity area index (S1 Fig).

To further analyze the effect of different sampling rates on the complexity of the LDF signal, we resampled the previously recorded blood flow signal and analyzed the complexity of the resampled signal. The results suggest that for the same segment of the blood flow signal time series, different sampling rates correspond to different MSE curves; however, the area index under the scale entropy curve remains relatively stable (S2 Fig). Therefore, the complexity area index can be used to evaluate the complexity of the LDF signal over a range of sampling rates.

**Fig 4:**
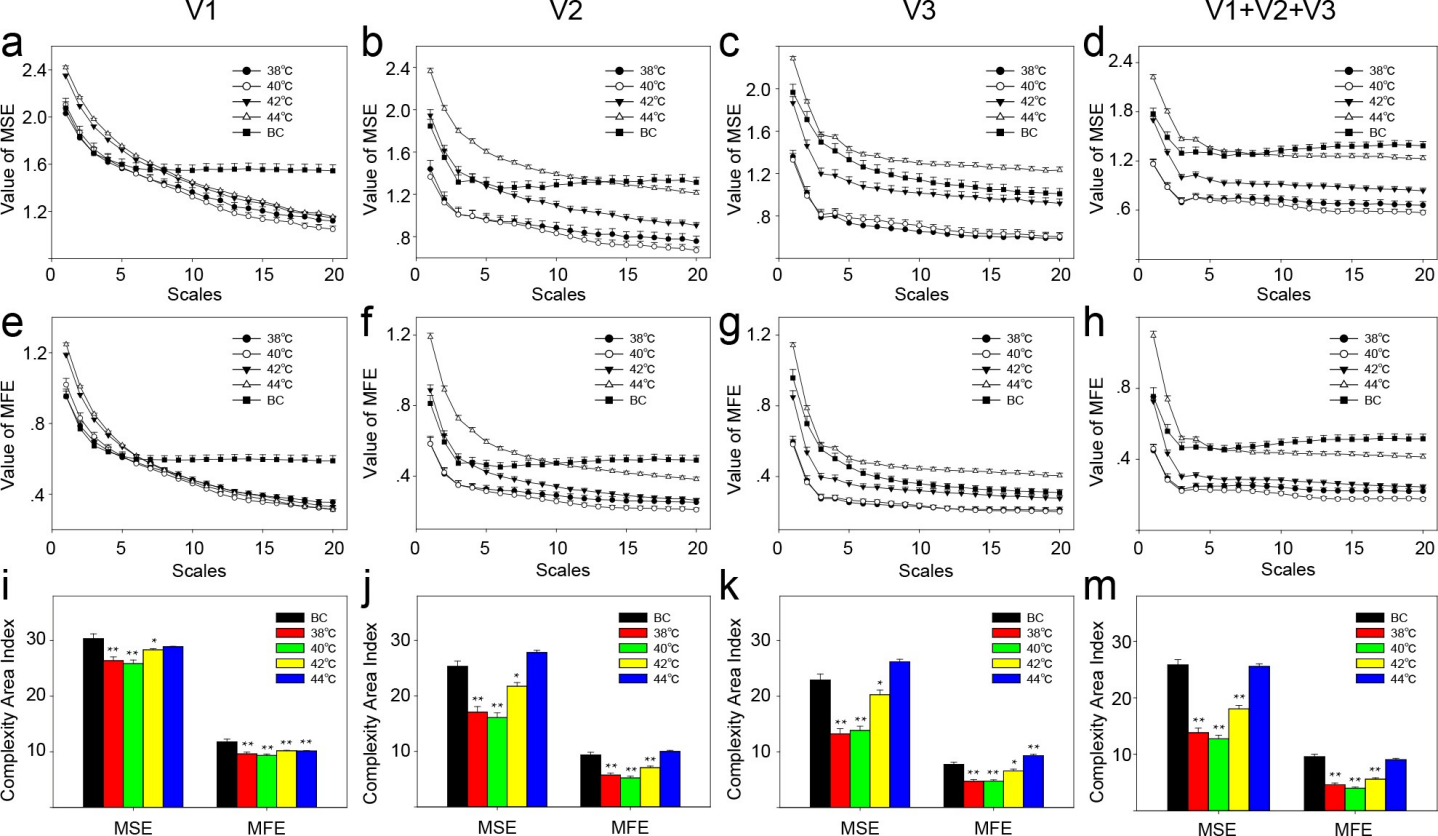
The complexity of speed-resolved perfusion at the stimulation point. (a) MSE result of the low-speed component (V1, <1 mm/s). (b) MSE result of the mid-speed component (V2, 1–10 mm/s). (c) MSE result of the high-speed component (V3, >10 mm/s). (d) MSE result of all speed components (V1+V2+V3). (e) MFE result of the low-speed component (V1, <1 mm/s). (f) MFE result of the mid-speed component (V2, 1–10 mm/s). (g) MFE result of the high-speed component (V3, >10 mm/s). (h) MFE result of all speed components (V1+V2+V3). (i) The complexity area index was obtained from the data of Fig 4A and Fig 4E. (j) Complexity area index obtained from the data of Fig 4B and Fig 4F. (k) Complexity area index obtained from the data of Fig 4C and Fig 4G. (m) Complexity area index obtained from the data of Fig 4D and Fig 4H. *, P<0.05; **, P<0.01, compared with BC, permutation test (two-sided, 1000 permutations). MSE, multiscale entropy; MFE; multiscale fuzzy entropy. BC, blank control; Data are presented as the mean±SE.

**Fig 5:**
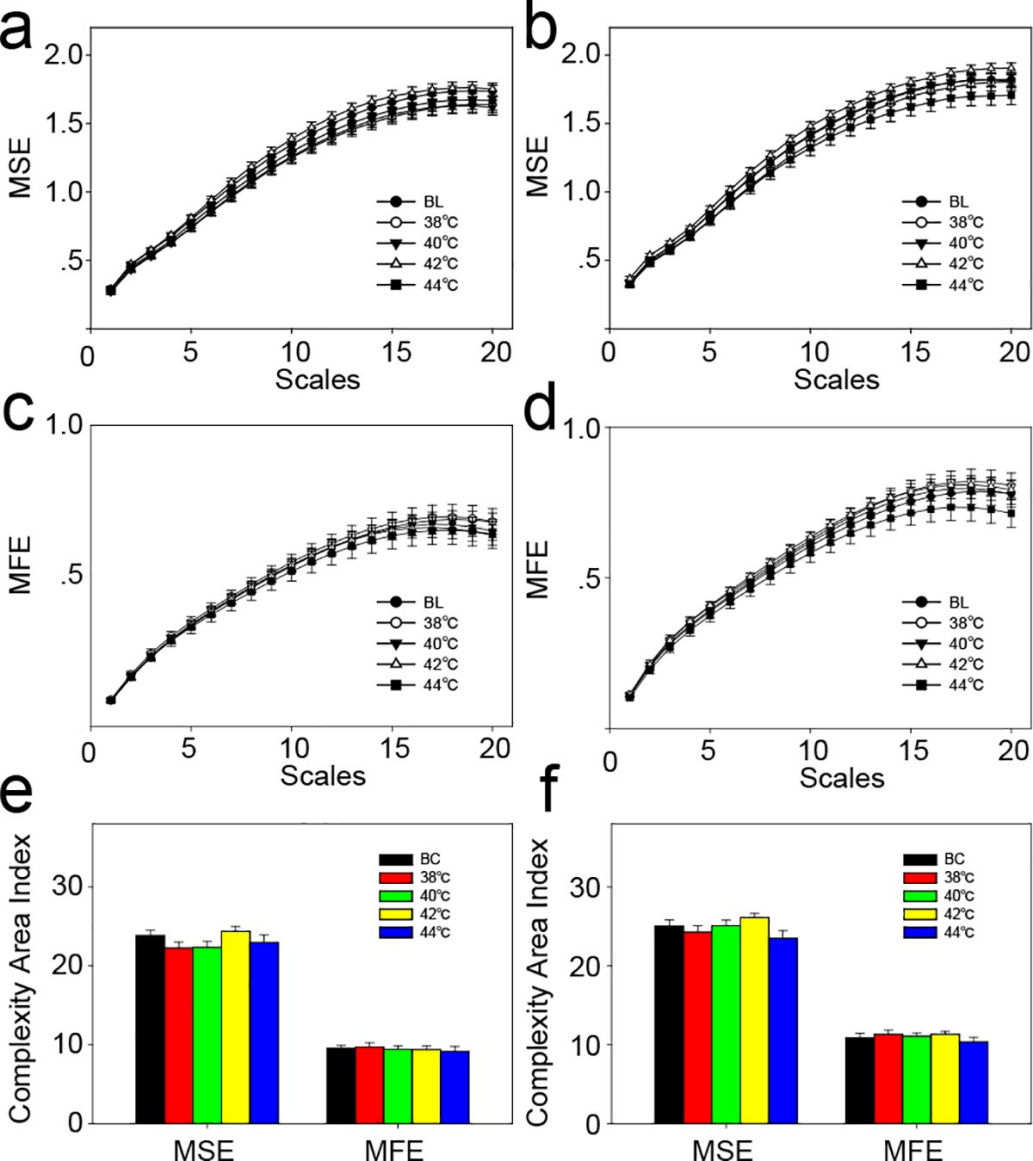
Complexity of blood perfusion at the control points. (a) MSE result of control point 1. (b) MSE result of control point 2. (c) MFE result of control point 1. (d) MFE result of control point 2. (e) Complexity area index obtained from the data of Fig 5A and Fig 5C. (f) Complexity area index obtained from the data of Fig 5B and Fig 4D. No significant difference was observed for the permutation test (two-sided, 1000 permutations). MSE, multiscale entropy; MFE; multiscale fuzzy entropy. BC, blank control; Data are presented as the mean±SE.

The complexity of each velocity component (V1, V2, and V3) and the composite component (V1+V2+V3) was analyzed using MSE and MFE. For MSE (Fig 4A, Fig 4B, Fig 4C and Fig 4D), 38°C or 40°C stimulation significantly reduced the complexity area index. As the temperature of the stimuli increased, the area index also increased. However, the complexity area index of the thermal stimulation group after 42°C stimulation was still smaller than that of the blank control group. When the stimulus temperature was increased to 44°C, the complexity area index showed no significant difference from that of the blank control group (Fig 4). The results for MFE (Fig 4E, Fig 4F, Fig 4G and Fig 4H) were similar to those for MSE. There were no changes in the complexity area index between control points 1 and 2 (Fig 5).

**Fig 6:**
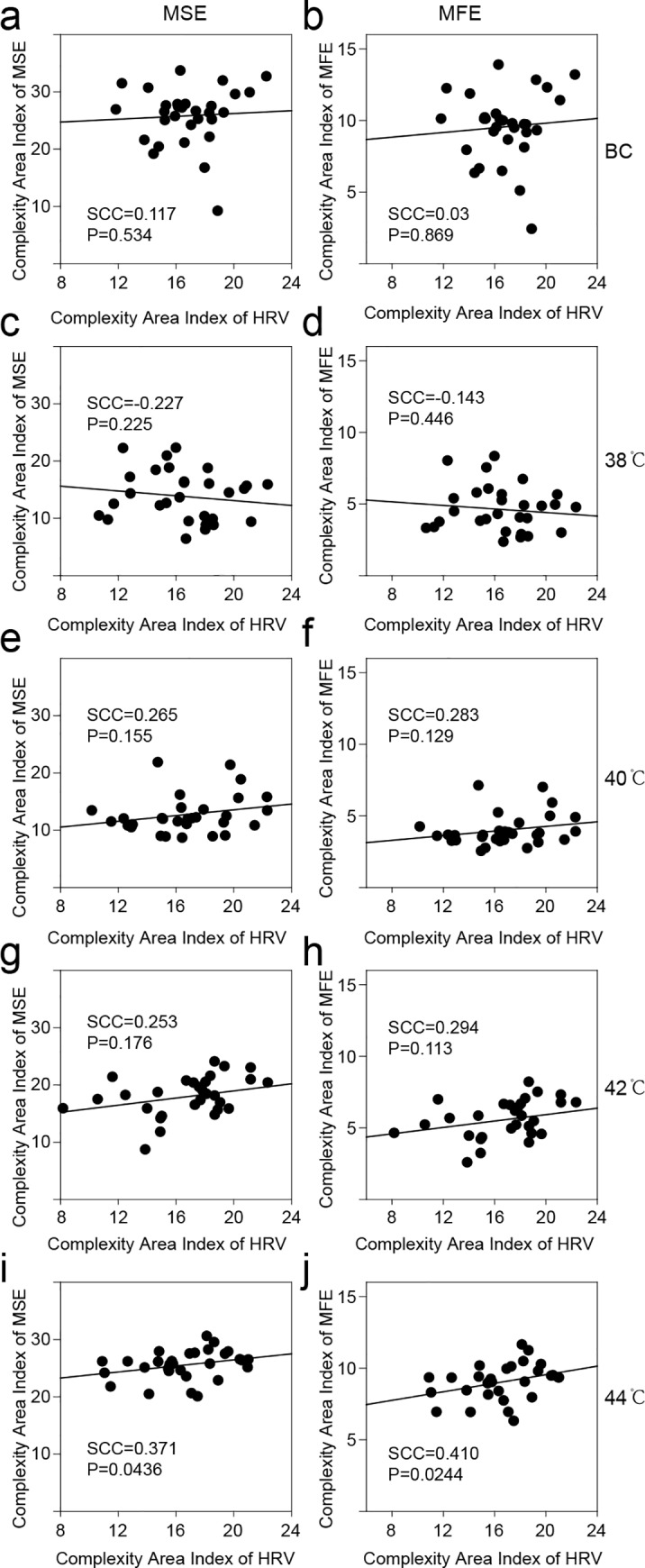
Relationship of the complexity between the composite blood perfusion (V1+V2+V3) and HRV. (a) MSE result of the blank control. (b) MFE result of the blank control. (c) MSE result of 38°C stimulation. (d) MFE result of 38°C stimulation. (e) MSE result of 40°C stimulation. (f) MFE result of 40°C stimulation. (g) MSE result of 42°C stimulation. (h) MFE result of 42°C stimulation. (i) MSE result of 44°C stimulation. (j) MFE result of 44°C stimulation. SCC, Spearman’s correlation coefficient; HRV, heart rate variability.

The analysis described here only evaluated the complexity of the blood flux signals at the stimulation site and at the control points. However, there were appropriate stimulation results in both local changes as well as a systemic response. A previous study indicated that the heart rate and the HRV can reflect the body’s response to an external stimulus. Consistent with previous studies[18], the results of the current study suggest that stimuli of different temperature also have no significant effect on the complexity of HRV (S3 Fig.). However, in contrast to previous research[18], there was a significant monotonic correlation between the composite signal (V1+V2+V3) and the HRV only at 44°C stimulation, not at 42°C stimulation (Fig 6). Moreover, this weak correlation did not appear when the V1 component (S4 Fig) and the V2 (S5 Fig) component were analyzed independently; a weak correlation appeared only between the MFE of the V3 component and HRV (S6 Fig).

## Discussion

In our previous study, classic LDF changes that were caused by stimulation with different temperatures were explored from the perspective of complexity. Furthermore, the relationship between the local blood flux and HRV was investigated[18]. However, local blood flow after stimulation with different temperatures from the perspective of blood flow distribution in different speed regions has not been evaluated previously. In the current study, the complexity of different velocity components was analyzed, which is an extension of our previous research. To the best of our knowledge, this is the first study to explore the relationship between speed-resolved perfusion and HRV complexity.

The complexity area index, which was proposed by Costa et al.[38], provides a more meaningful measure of complexity. It can demonstrate the structural richness of information by estimating the area under MSE curves (S7 Fig). In other words, the sum of entropy values for scales was defined as the complexity area index. A previous study indicated that the decreased complexity area index of the special channel EEG was associated with epilepsy, which suggested that the complexity area index might be a potential predictor to clarify who will be more likely to have later epilepsy[39]. Another study revealed that the MSE complexity area index was reduced in patients with diabetes[40]. All of these results suggest that the complexity area index can be widely used as a biomarker for judging the human state[41]. Of course, in specific applications, the special area index of different scales, such as from scale 1 to scale 5[42], can be calculated. In the current study, the area index of all scales from 1 to 20 was calculated.

According to previous studies, blood perfusion signals recorded by LDF can be separated into different frequency bands in the frequency domain[43–46]; these frequency bands might reflect different physiological rhythms[47]. From another perspective, microcirculation perfusion can also be distinguished by the speed distribution, and the speed of red blood cells (RBCs) is closely related to the total cross-sectional area of different types of blood vessels. Therefore, different velocity components have potential value for distinguishing different types of blood vessels, thereby exploring the regulatory mechanisms of local microcirculation. Our current research suggests that, regardless of the speed-resolved component, microcirculation perfusion is significantly increased after thermal stimulation. However, there is no direct correlation between the increase in blood perfusion that is caused by thermal stimulation and the complexity of blood flow signals. This result is consistent with the results of classic LDF signals[18]. By applying computational models, the EPOS system enables absolute measures of tissue volume that contain different vessel structures as capillaries, venules and arterioles. By separating the perfusion into speed regions, the different vascular effects that resulted from thermal stimulation can be studied separately. Therefore, our results provide an opportunity for deeper insight into the endothelial and neurovascular function and for further understanding of how microcirculation responds to different types of thermal stimulation in human skin microcirculation.

Previous results indicated that a multiscale analysis of blood flux has the potential to distinguish between the different hemodynamic states[48]. When assessed using MSE, the microvascular blood flow of healthy human skin was very sensitive to the changes induced by local warming. Although the complexity of the blood flow and the oxygen saturation signals were analyzed simultaneously, it did not monitor the control point flux and HRV. A systemic response to appropriate temperature stimulation was not observed from the perspective of complexity. From the current study, lower temperature stimuli can change the complexity of the local blood flux. However, higher temperature stimuli do not alter the complexity of the adjacent blood flux and HRV, which suggests that systemic thresholds are usually higher than local thresholds. Local supra-threshold stimulation does not lead to global reactions, which benefits the maintenance of the stability of the internal environment.

In this study, the complexity of the HRV was calculated by using MSE to assess the thermal effect. The results suggested that below 44°C stimulation, the complexity area index of the HRV had no significant changes. However, a correlation analysis indicated that when the temperature stimulus was 44°C, the complexity of the composite blood flux signal was weakly related to HRV.

In the current study, only temperature stimuli from 38°C to 44°C were applied, and nociceptive thermal stimulation was not observed. Although from our analysis results, the complexity area index that corresponded to different sampling rates was relatively stable, this was only the result of classic LDF. Due to the limitation of the EPOS sampling rate, the influence of different sampling frequencies on the complexity of speed-resolved blood perfusion has not been further analyzed, which is also a limitation of our research. In addition, because the main purpose of the current study was to observe the effects of stimulation with different temperatures on the speed-resolved components, the effect of the acupoint specificity was not addressed.

Since measurements are not carried out at a fixed time, we cannot negate the effect of the circadian rhythm on our outcome, which is also a shortcoming of the current study.

## Conclusions

Different complexity area indexes of speed-resolved blood perfusion were observed following different thermal stimulations. A monotonic correlation of the complexity area index between HRV and both the high-velocity and the composite local blood components was found after 44°C thermal stimulation. The current study provides an approach to explore the relationship between the heart function and the acupoint velocity-resolved perfusion via a complexity analysis.

## Supporting information

S1 Table Thermal stimulation order and experiment interval days of each subject. (DOCX)

S2 Table Subject’s heart rate (HR) before thermal stimulation. (DOCX)

S1 Fig MSE and MFE result of white noise signals with different sampling rates. The values of MSE and MFE were both derived from white noise signals with a data length of 96000. (a) MSE result. (b) MFE result. (c) Complexity area index obtained from the data of S1A and S1B Fig. No significant difference, permutation test (two-sided, 1000 permutations). MSE, multiscale entropy; MFE, multiscale fuzzy entropy. Data are presented as the mean±SE.
(TIF)

S2 Fig MSE and MFE results of classic LFD signals with different sampling rates. All data are derived from a previous study (Wang, G, *et al*. Scientific Reports 8, 8982). (a) MSE result for a 32-Hz sampling rate. (b) MFE result for a 32-Hz sampling rate. (c) Complexity area index for a 32-Hz sampling rate obtained from the data of S2A and S2B Fig. (d) MSE result for a 16-Hz sampling rate. (e) MFE result for a 16-Hz sampling rate. (f) Complexity area index for a 16-Hz sampling rate obtained from the data of S2D and S2E Fig. (g) MSE result for an 8-Hz sampling rate. (h) MFE result for an 8-Hz sampling rate. (i) Complexity area index for an 8-Hz sampling rate obtained from the data of S2G and S2H Fig. (j) MSE result for a 4-Hz sampling rate. (k) MFE result for a 4-Hz sampling rate. (l) Complexity area index for a 4-Hz sampling rate obtained from the data of S2J and S2K Fig. (m) MSE result for a 3-Hz sampling rate. (n) MFE result for a 3-Hz sampling rate. (o) Complexity area index for a 3-Hz sampling rate obtained from the data of S2M and S2N Fig. *, P<0.05; **, P<0.01, compared with BL, permutation test (two-sided, 1000 times permutation). MSE, multiscale entropy; MFE; multiscale fuzzy entropy. BL, baseline; Data are presented as the mean±SE.
(TIF)

S3 Fig Complexity of the HRV. **(a) Raw ECG data. (b) RR intervals of an ECG.** (c) Complexity of the HRV under different thermal stimuli. (d) Complexity area index of different conditions. P>0.05. Data are presented as the mean±SE.
(TIF)

S4 Fig Relationship of the complexity between V1 (low speed, <1 mm/s) and HRV. (a) MSE result of the blank control. (b) MFE result of the blank control. (c) MSE result of 38°C stimulation. (d) MFE result of 38°C stimulation. (e) MSE result of 40°C stimulation. (f) MFE result of 40°C stimulation. (g) MSE result of 42°C stimulation. (h) MFE result of 42°C stimulation. (i) MSE result of 44°C stimulation. (j) MFE result of 44°C stimulation. SCC, Spearman’s correlation coefficient.
(TIF)

S5 Fig Relationship of the complexity between V2 (mid-speed, 1–10 mm/s) and HRV. (a) MSE result of the blank control. (b) MFE result of the blank control. (c) MSE result of 38°C stimulation. (d) MFE result of 38°C stimulation. (e) MSE result of 40°C stimulation. (f) MFE result of 40°C stimulation. (g) MSE result of 42°C stimulation. (h) MFE result of 42°C stimulation. (i) MSE result of 44°C stimulation. (j) MFE result of 44°C stimulation. SCC, Spearman’s correlation coefficient.
(TIF)

S6 Fig Relationship of the complexity between V3 (high speed, >10 mm/s) and HRV. (a) MSE result of the blank control. (b) MFE result of the blank control. (c) MSE result of 38°C stimulation. (d) MFE result of 38°C stimulation. (e) MSE result of 40°C stimulation. (f) MFE result of 40°C stimulation. (g) MSE result of 42°C stimulation. (h) MFE result of 42°C stimulation. (i) MSE result of 44°C stimulation. (j) MFE result of 44°C stimulation. SCC, Spearman’s correlation coefficient.
(TIF)

S7 Fig Diagram of the complexity area index. (TIF)

S1 File Method of the MSE and MFE analysis. (DOCX)
