# Supplementary material for: Exploring the relationship between the speed-resolved perfusion of blood flux and HRV following different thermal stimulations using MSE and MFE analyses
Source: PLoS One. 2019 Jun 5;14(6):e0217973. doi: 10.1371/journal.pone.0217973 (PMC6550418; doi:10.1371/journal.pone.0217973)
Supplement: S1 Table — (DOCX) [file pone.0217973.s001.docx]

| Subject NO. | 1^st^  （℃） | Interval  （days） | 2^nd^  （℃） | Interval  （days） | 3^th^  （℃） | Interval  （days） | 4^th^  （℃） |
| --- | --- | --- | --- | --- | --- | --- | --- |
| 1 | 44 | 8 | 40 | 8 | 42 | 16 | 38 |
| 2 | 44 | 25 | 38 | 8 | 40 | 18 | 42 |
| 3 | 38 | 34 | 44 | 15 | 40 | 14 | 42 |
| 4 | 42 | 23 | 40 | 20 | 44 | 11 | 38 |
| 5 | 42 | 18 | 38 | 8 | 40 | 13 | 44 |
| 6 | 38 | 27 | 42 | 16 | 44 | 17 | 40 |
| 7 | 38 | 37 | 44 | 12 | 42 | 11 | 40 |
| 8 | 38 | 26 | 40 | 24 | 42 | 8 | 44 |
| 9 | 38 | 28 | 42 | 14 | 44 | 9 | 40 |
| 10 | 42 | 27 | 44 | 8 | 38 | 15 | 40 |
| 11 | 40 | 27 | 38 | 8 | 42 | 7 | 44 |
| 12 | 38 | 27 | 44 | 8 | 40 | 7 | 42 |
| 13 | 42 | 29 | 44 | 13 | 40 | 7 | 38 |
| 14 | 42 | 29 | 40 | 14 | 44 | 12 | 38 |
| 15 | 38 | 33 | 42 | 22 | 40 | 9 | 44 |
| 16 | 42 | 24 | 40 | 10 | 38 | 13 | 44 |
| 17 | 40 | 8 | 44 | 7 | 38 | 8 | 42 |
| 18 | 38 | 14 | 40 | 13 | 42 | 6 | 44 |
| 19 | 40 | 22 | 44 | 7 | 38 | 8 | 42 |
| 20 | 44 | 8 | 40 | 6 | 42 | 15 | 38 |
| 21 | 42 | 16 | 38 | 8 | 44 | 7 | 40 |
| 22 | 42 | 6 | 40 | 8 | 38 | 16 | 44 |
| 23 | 42 | 14 | 44 | 7 | 38 | 7 | 40 |
| 24 | 42 | 10 | 44 | 19 | 38 | 10 | 40 |
| 25 | 42 | 12 | 38 | 8 | 40 | 8 | 44 |
| 26 | 42 | 14 | 38 | 13 | 40 | 8 | 44 |
| 27 | 40 | 19 | 44 | 7 | 42 | 8 | 38 |
| 28 | 44 | 13 | 40 | 10 | 38 | 8 | 42 |
| 29 | 42 | 5 | 38 | 13 | 40 | 7 | 44 |
| 30 | 40 | 7 | 42 | 6 | 38 | 7 | 44 |

S1 Table. Thermal stimulation order and intervals of each subject
