# Supplementary material for: Exploring the relationship between the speed-resolved perfusion of blood flux and HRV following different thermal stimulations using MSE and MFE analyses
Source: PLoS One. 2019 Jun 5;14(6):e0217973. doi: 10.1371/journal.pone.0217973 (PMC6550418; doi:10.1371/journal.pone.0217973)
Supplement: S2 Table — (DOCX) [file pone.0217973.s002.docx]

|  | BC | 38℃ | 40℃ | 42℃ | 44℃ |
| --- | --- | --- | --- | --- | --- |
| HR | 67.27±8.12 | 67.68±8.11 | 69.12±9.57 | 68.25±10.15 | 67.22±8.71 |

S2 Table. Heart rate (HR) of subject before thermal stimulation

There was no significant difference between groups.
