# Supplementary figures and images for: Exploring the relationship between the speed-resolved perfusion of blood flux and HRV following different thermal stimulations using MSE and MFE analyses
Source: PLoS One. 2019 Jun 5;14(6):e0217973. doi: 10.1371/journal.pone.0217973 (PMC6550418; doi:10.1371/journal.pone.0217973)

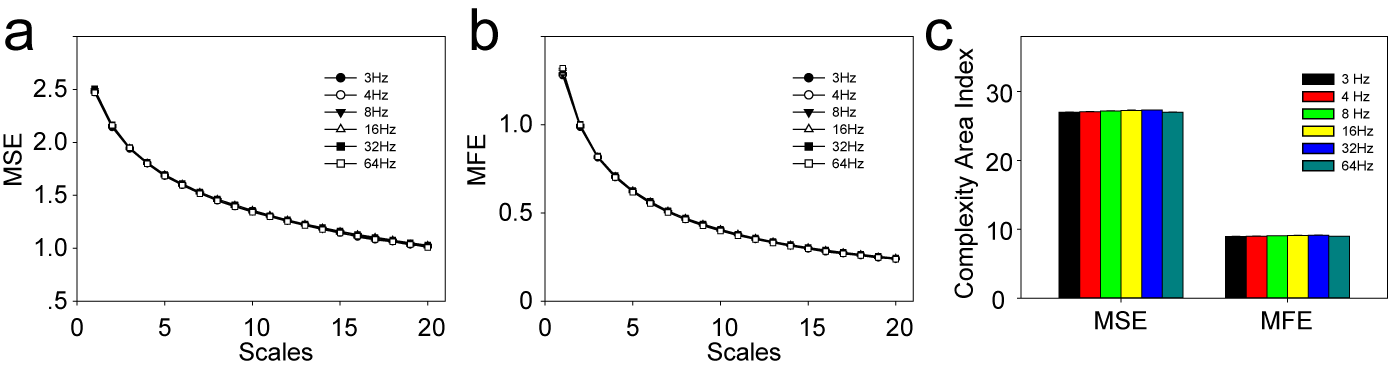

Supplement: S1 Fig — The values of MSE and MFE were both derived from white noise signals with a data length of 96000. (a) MSE result. (b) MFE result. (c) Complexity area index obtained from the data of S1A and S1B Fig. No significant difference, permutation test (two-sided, 1000 permutations). MSE, multiscale entropy; MFE, multiscale fuzzy entropy. Data are presented as the mean±SE. (TIF) [file pone.0217973.s003.tif]

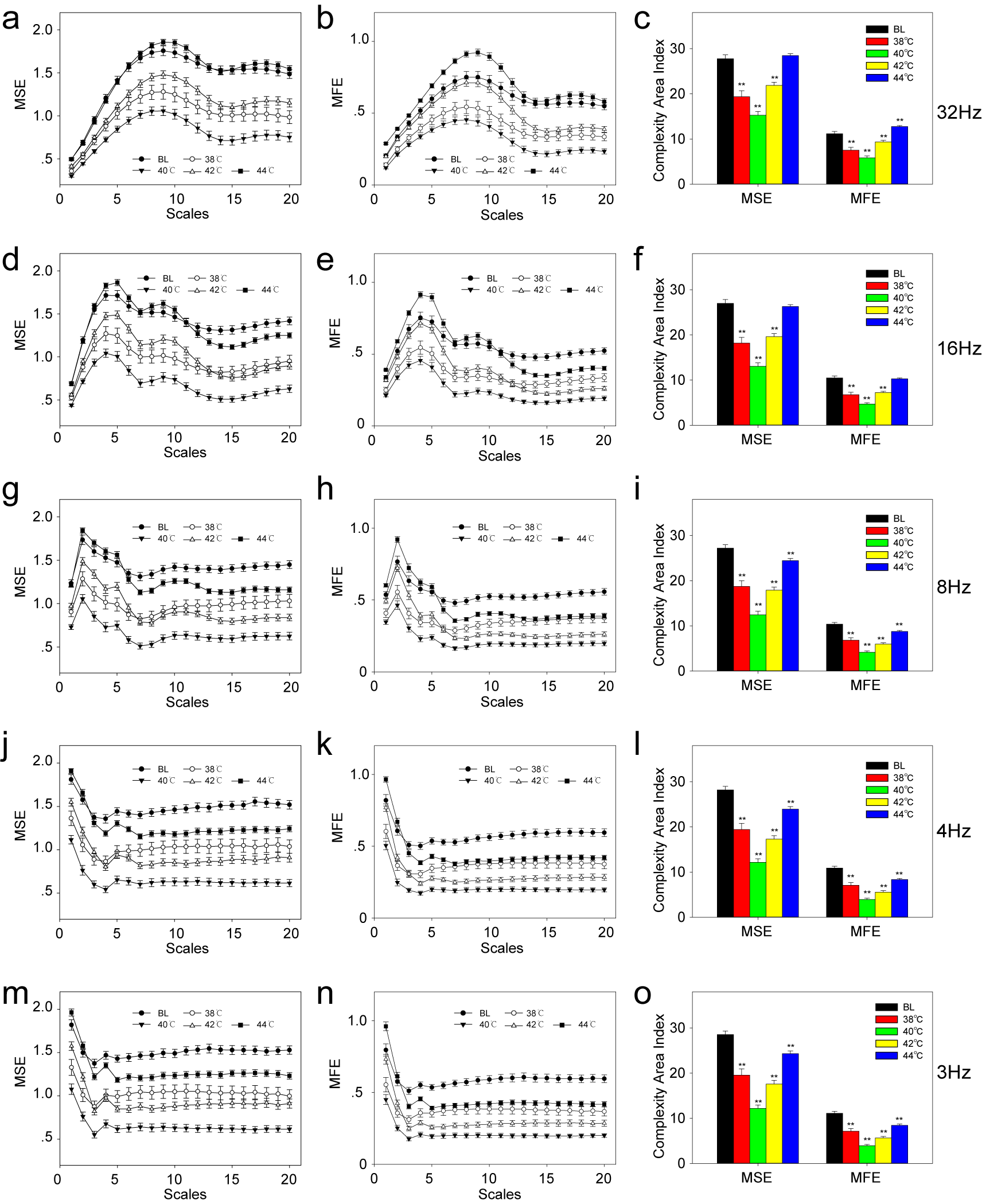

Supplement: S2 Fig — All data are derived from a previous study (Wang, G, et al. Scientific Reports 8, 8982). (a) MSE result for a 32-Hz sampling rate. (b) MFE result for a 32-Hz sampling rate. (c) Complexity area index for a 32-Hz sampling rate obtained from the data of S2A and S2B Fig. (d) MSE result for a 16-Hz sampling rate. (e) MFE result for a 16-Hz sampling rate. (f) Complexity area index for a 16-Hz sampling rate obtained from the data of S2D and S2E Fig. (g) MSE result for an 8-Hz sampling rate. (h) MFE result for an 8-Hz sampling rate. (i) Complexity area index for an 8-Hz sampling rate obtained from the data of S2G and S2H Fig. (j) MSE result for a 4-Hz sampling rate. (k) MFE result for a 4-Hz sampling rate. (l) Complexity area index for a 4-Hz sampling rate obtained from the data of S2J and S2K Fig. (m) MSE result for a 3-Hz sampling rate. (n) MFE result for a 3-Hz sampling rate. (o) Complexity area index for a 3-Hz sampling rate obtained from the data of S2M and S2N Fig. *, P<0.05; **, P<0.01, compared with BL, permutation test (two-sided, 1000 times permutation). MSE, multiscale entropy; MFE; multiscale fuzzy entropy. BL, baseline; Data are presented as the mean±SE. (TIF) [file pone.0217973.s004.tif]

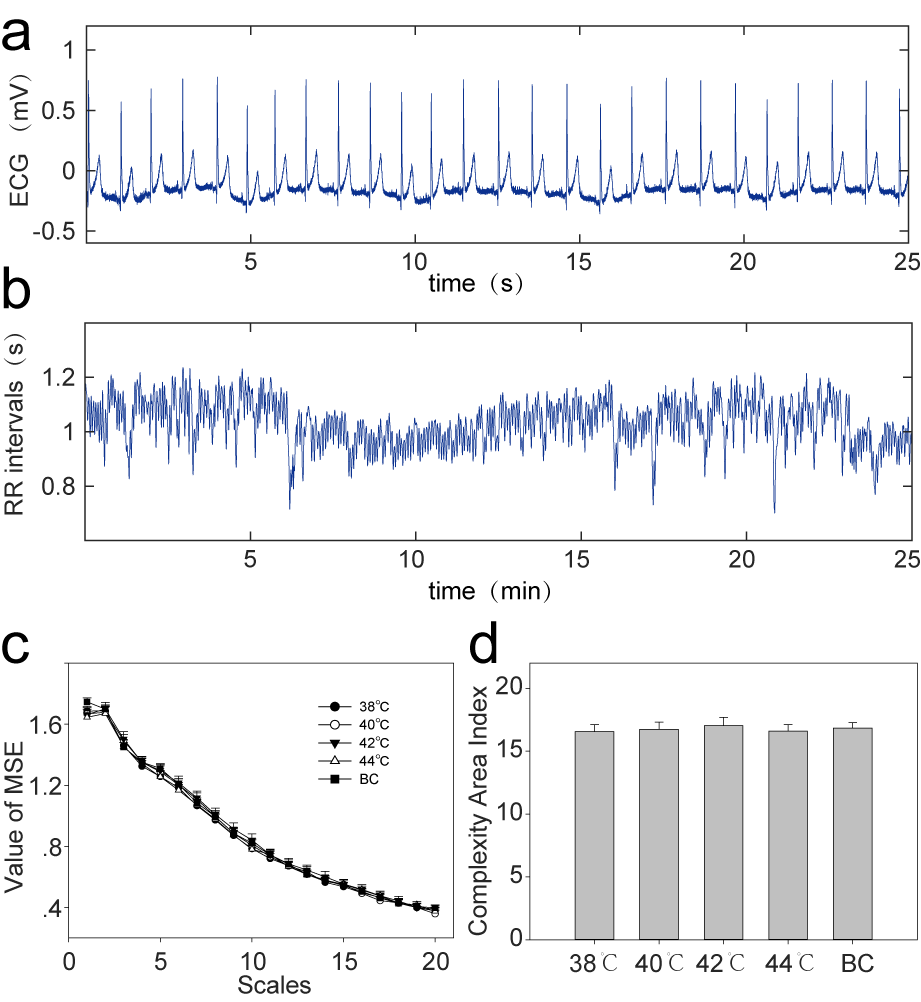

Supplement: S3 Fig — (a) Raw ECG data. (b) RR intervals of an ECG. (c) Complexity of the HRV under different thermal stimuli. (d) Complexity area index of different conditions. P>0.05. Data are presented as the mean±SE. (TIF) [file pone.0217973.s005.tif]

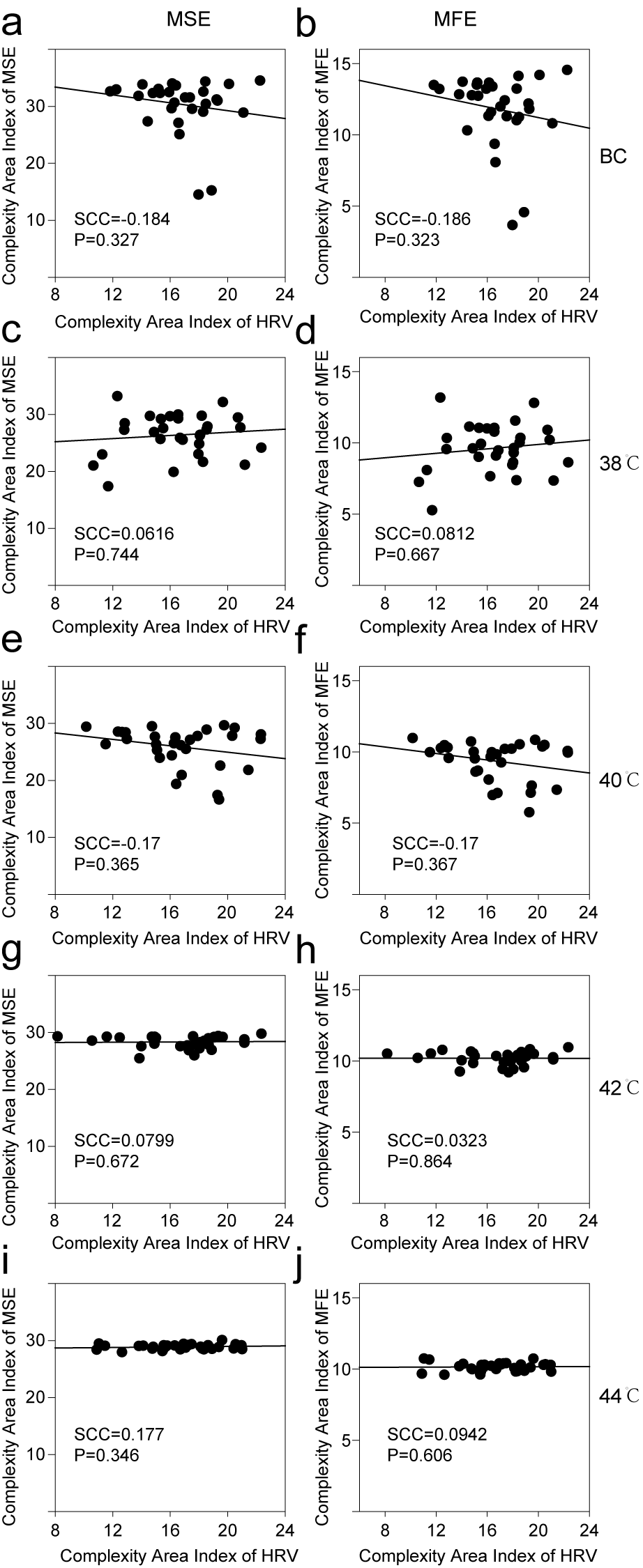

Supplement: S4 Fig — (a) MSE result of the blank control. (b) MFE result of the blank control. (c) MSE result of 38°C stimulation. (d) MFE result of 38°C stimulation. (e) MSE result of 40°C stimulation. (f) MFE result of 40°C stimulation. (g) MSE result of 42°C stimulation. (h) MFE result of 42°C stimulation. (i) MSE result of 44°C stimulation. (j) MFE result of 44°C stimulation. SCC, Spearman’s correlation coefficient. (TIF) [file pone.0217973.s006.tif]

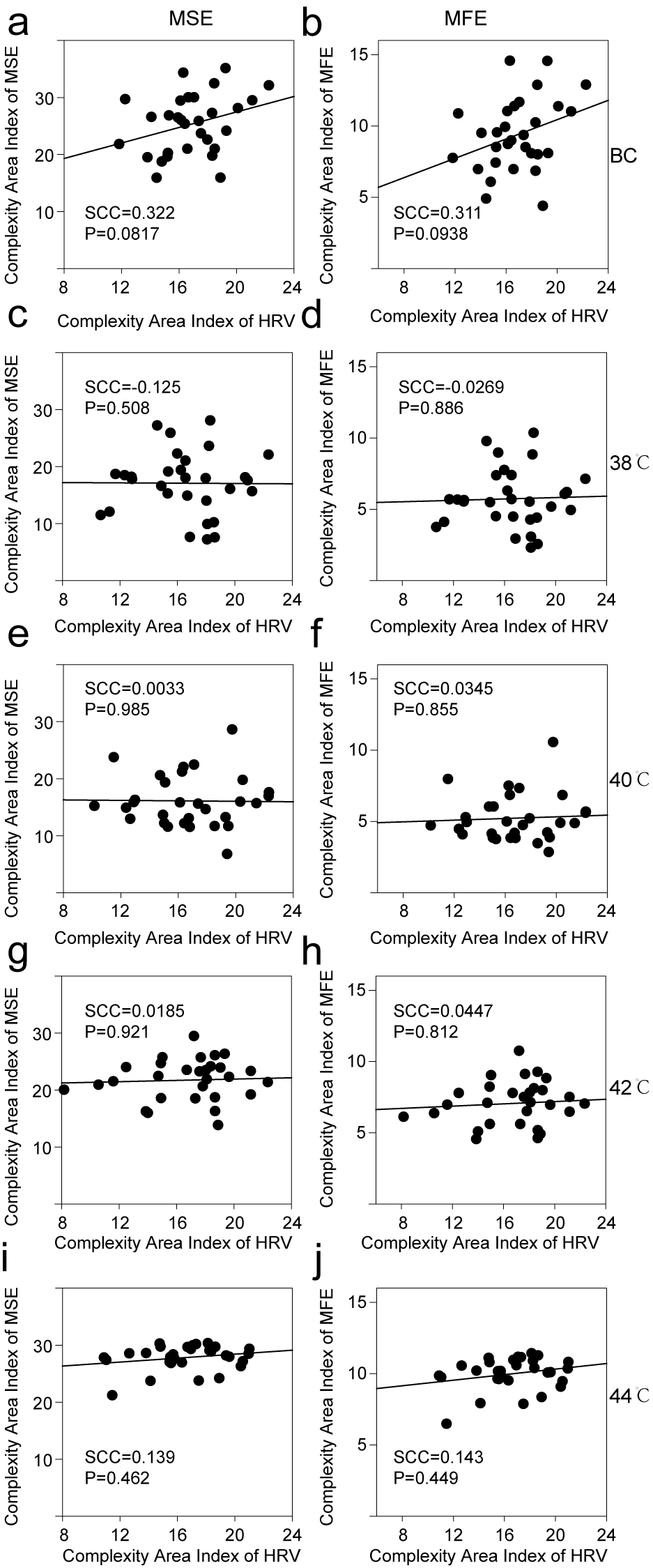

Supplement: S5 Fig — (a) MSE result of the blank control. (b) MFE result of the blank control. (c) MSE result of 38°C stimulation. (d) MFE result of 38°C stimulation. (e) MSE result of 40°C stimulation. (f) MFE result of 40°C stimulation. (g) MSE result of 42°C stimulation. (h) MFE result of 42°C stimulation. (i) MSE result of 44°C stimulation. (j) MFE result of 44°C stimulation. SCC, Spearman’s correlation coefficient. (TIF) [file pone.0217973.s007.tif]

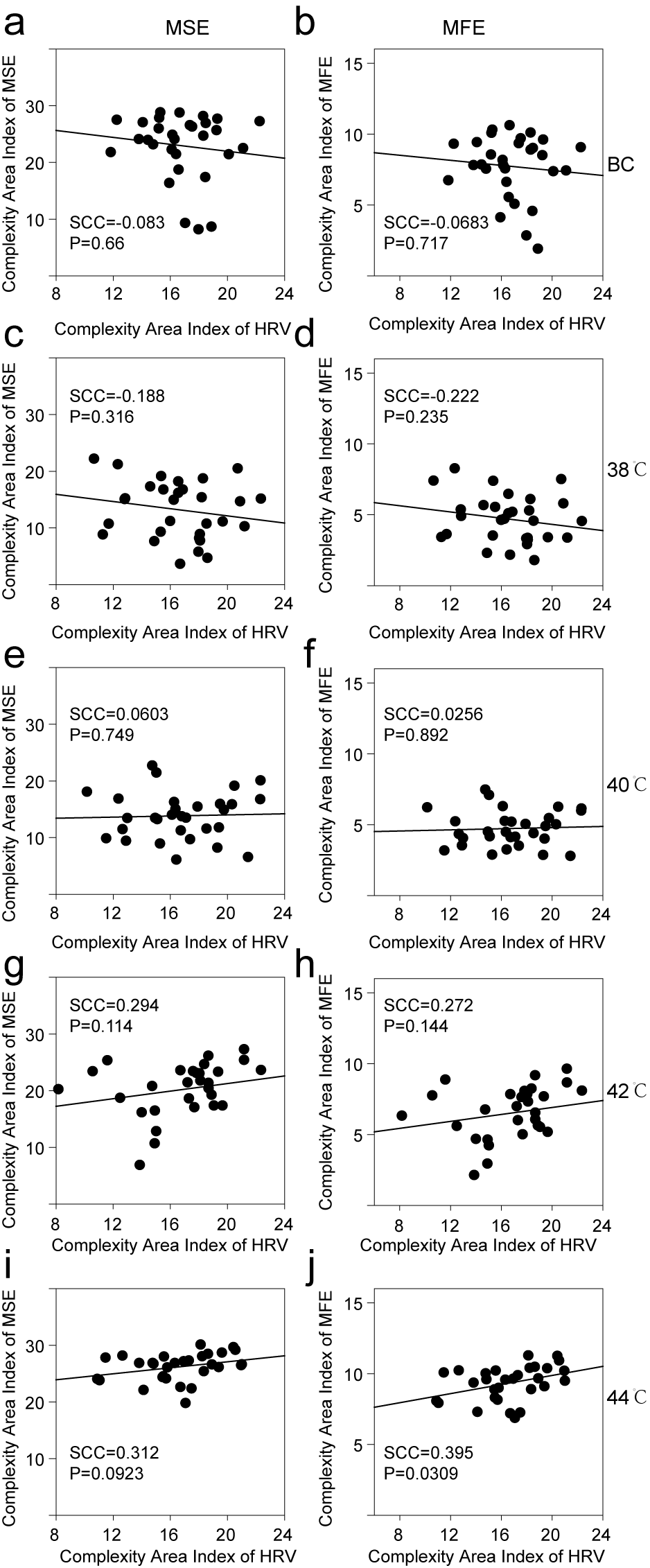

Supplement: S6 Fig — (a) MSE result of the blank control. (b) MFE result of the blank control. (c) MSE result of 38°C stimulation. (d) MFE result of 38°C stimulation. (e) MSE result of 40°C stimulation. (f) MFE result of 40°C stimulation. (g) MSE result of 42°C stimulation. (h) MFE result of 42°C stimulation. (i) MSE result of 44°C stimulation. (j) MFE result of 44°C stimulation. SCC, Spearman’s correlation coefficient. (TIF) [file pone.0217973.s008.tif]

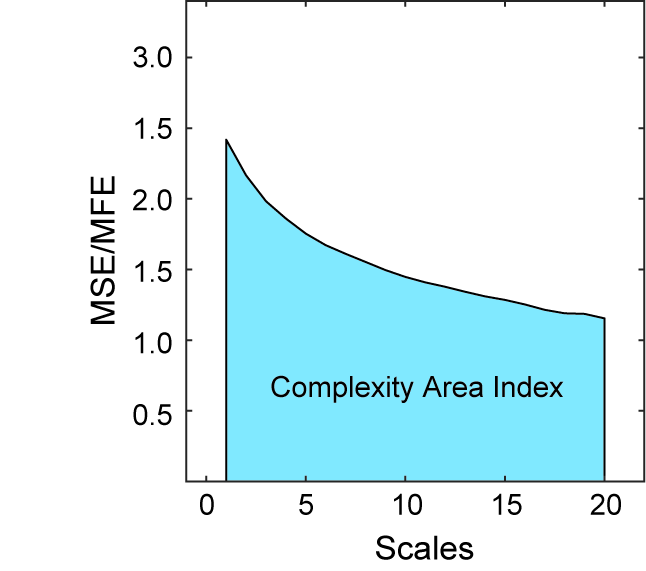

Supplement: S7 Fig — (TIF) [file pone.0217973.s009.tif]
