# Supplementary material for: Exploring the relationship between the speed-resolved perfusion of blood flux and HRV following different thermal stimulations using MSE and MFE analyses
Source: PLoS One. 2019 Jun 5;14(6):e0217973. doi: 10.1371/journal.pone.0217973 (PMC6550418; doi:10.1371/journal.pone.0217973)
Supplement: S1 File — (DOCX) [file pone.0217973.s010.docx]

**Analysis for MSE**

For the original time series $\{x_{i}\}$, $(i=1,2,\cdots\cdots,N)$, The $m$ dimensional vector is constructed as $X(i)=\{x(i),x(i+1),\cdots,x(i+m-1)\}(i=1,2,\cdots,N-m+1)$. The max distance between $X(i)$ and $X(j)$ were defined as:

$$d[X(i),X(j)]=\mathop{Max}\limits_{k=0\to m-1} \left\{\left|X(i+k)-X(j+k)\right|\right\}$$

For each $i$, the max distance between$X(i)$ and $X(j)$ ,$(i=1,2,\cdots N-m+1,j=1,2,\cdots N-m+1,j\neq i)$ is calculated, then the$d_{m}[X(i),X(j)]$is obtained. $r$ is the tolerance for accepting matches. $C_{i}^{m}(r)$ is the ratio of the number of $d_{m}[X(i),X(j)]<r$ to total distance.

$$C_{i}^{m}(r)=\frac{1}{N-m}num\left\{d_{m}[X(i),X(j)]<r\right\}$$

$C^{m}(r)$is the average of $C_{i}^{m}(r)$

$$C^{m}(r)=\frac{1}{N-m+1}\sum_{i=1}^{N-m+1} C_{i}^{m}(r)$$

While from $m$ to $m+1$, $C^{m+1}(r)$ is calculated again. The sample entropy (SampEn) is defined below:

$$SampEn(m,r,N)=-\ln\frac{C^{m+1}(r)}{C^{m}(r)}$$

For MSE analysis, the original time series $\{x_{i}\},(i=1,2,\cdots\cdots,N)$ were coarse-grained according to the equation.

$$y_{j}^{(\tau)}=\frac{1}{\tau}\sum_{i=(j-1)\tau+1}^{j\tau} x_{i}\left(1\leq j\leq\frac{N}{\tau}\right)$$

Where $\tau$ represents the scale-factor ranged from 1 to 20 in current study, and then calculated sampEn of each coarse-grained time series.

**Analysis for MFE**

Given an $N$ sample time series $\{x_{i}\}$, $(i=1,2,\cdots\cdots,N)$, The $m$ dimensional vector is constructed as $X(i)=\{x(i),x(i+1),\cdots,x(i+m-1)\}-x_{0}(i),(i=1,2,\cdots,N-m+1)$. Where $x_{0}(i)$ is the average of vector $X(i)$. The max distance between $X(i)$ and $X(j)$ were defined as:

$$d\left[X(i),X(j)\right]=\mathop{Max}\limits_{k=0\to m-1} \left\{\left|(X(i+k)-x_{0}(i))-(X(j+k)-x_{0}(j))\right|\right\}$$

For each $i$, the max distance between$X(i)$ and $X(j)$ ,$(i=1,2,\cdots N-m+1,j=1,2,\cdots N-m+1,j\neq i)$ is calculated, then the $d_{m}\left[X(i),X(j)\right]$is obtained. Give $n$ and $r$, the similarity degree $D_{ij}^{m}$ was calculated.

$$D_{ij}^{m}=e^{\left(\frac{-(d_{m}[X(i),X(j)])^{n}}{r}\right)}$$

Define the function$C^{m}(n,r)$ as follows:

$$C^{m}(n,r)=\frac{1}{N-m}\sum_{i=1}^{N-m} \left(\frac{1}{N-m+1}\sum_{j=1,j\neq i}^{N-m} D_{ij}^{m}\right)\text{=}\frac{1}{N-m}\sum_{i=1}^{N-m} \left(\frac{1}{N-m+1}\sum_{j=1,j\neq i}^{N-m} e^{\left(\frac{-(d_{m}[X(i),X(j)])^{n}}{r}\right)}\right)$$

While from $m$ to $m+1$, $C^{m+1}(n,r)$ is calculated. The Fuzzy entropy (FuzzyEn) is defined below:

$$FuzzyEn(m,n,r,N)=-\ln\frac{C^{m+1}(n,r)}{C^{m}(n,r)}$$

For MFE analysis, the original time series $\{x_{i}\},(i=1,2,\cdots\cdots,N)$ were coarse-grained according to the equation.

$$y_{j}^{(\tau)}=\frac{1}{\tau}\sum_{i=(j-1)\tau+1}^{j\tau} x_{i}\left(1\leq j\leq\frac{N}{\tau}\right)$$

Where $\tau$ represents the scale-factor ranged from 1 to 20 in current study, and then calculated FuzzyEn of each coarse-grained time series.
